# COVID-19 Outbreak Among College Students After a Spring Break Trip to Mexico — Austin, Texas, March 26–April 5, 2020

**DOI:** 10.15585/mmwr.mm6926e1

**Published:** 2020-07-03

**Affiliations:** ^1^Dell Medical School, University of Texas at Austin, Texas; ^2^University of Texas Health Sciences Center, School of Public Health at Austin, Texas; ^3^Austin Public Health, Austin, Texas.

*On June 24, 2020, this report was posted online as an *MMWR* Early Release.*

On March 27, 2020, a University of Texas at Austin student with cough, sore throat, and shortness of breath had a positive test result for SARS-CoV-2, the virus that causes coronavirus disease 2019 (COVID-19). On March 28, two more symptomatic students had positive test results, alerting the COVID-19 Center at the University of Texas Health Austin (UTHA) to a potential outbreak; the center initiated an outbreak investigation the same day. UTHA conducted contact tracing, which linked the students’ infections to a spring break trip to Cabo San Lucas, Mexico, during March 14–19. Among 231 persons tested for SARS-CoV-2 in this investigation, 64 (28%) had positive test results, including 60 (33%) of 183 Cabo San Lucas travelers, one of 13 (8%) household contacts of Cabo San Lucas travelers, and three (9%) of 35 community contacts of Cabo San Lucas travelers. Approximately one fifth of persons with positive test results were asymptomatic; no persons needed hospitalization, and none died. This COVID-19 outbreak among a young, healthy population with no or mild symptoms was controlled with a coordinated public health response that included rapid contact tracing and testing of all exposed persons. A coordinated response with contact tracing and testing of all contacts, including those who are asymptomatic, is important in controlling future COVID-19 outbreaks that might occur as schools and universities consider reopening.

## Investigation and Results

During March 27–28, three symptomatic University of Texas students had positive test results for SARS-CoV-2. All three had traveled to Cabo San Lucas, Mexico, during March 14–19 and became symptomatic after returning (March 22–25). On March 28, the UTHA COVID-19 Center, a multidisciplinary team established in early March to conduct testing, contact tracing, and monitoring for the University of Texas community with authority delegated from Austin Public Health, initiated an investigation. Additional travelers were identified through contact tracing interviews and review of flight manifests gathered with assistance from Austin Public Health. Travelers on chartered or private flights were traced by UTHA and any potential commercial flight exposures were escalated through Austin Public Health to the Texas Department of State Health Services. Travelers and contacts of any travelers with a positive SARS-CoV-2 test result were classified into one of three categories: Cabo San Lucas travelers (i.e., persons who traveled to Cabo San Lucas), household contacts (i.e., persons who did not travel to Cabo San Lucas, but who lived with a Cabo San Lucas traveler who had a positive test result), or community contacts (i.e., persons who did not travel to Cabo San Lucas, but who had close contact in a community setting to a Cabo San Lucas traveler who had a positive test result). A case was defined as a positive SARS-CoV-2 reverse transcription–polymerase chain reaction (RT-PCR) test result in any traveler to Cabo San Lucas during March 14–19 or any of the travelers’ household or community contacts identified during March 19–April 2.

With oversight from a university epidemiologist and infectious diseases physician, UTHA trained medical students, public health students, and clinical and research staff members to trace contacts. UTHA contact tracers communicated with travelers and contacts by telephone, first texting an initial message about the potential exposure and then attempting to call each traveler and contact up to three times. Through interviews with travelers and contacts, the date and method of return travel (i.e., commercial or charter flight and flight number for those who traveled to Cabo San Lucas), date of last exposure to a patient with known COVID-19, presence of symptoms, symptom onset date, and current address were collected and recorded. For those travelers and contacts without symptoms, the date of testing was used as a proxy for symptom onset date to estimate an infectious period. During the telephone call, contact tracers advised asymptomatic travelers and contacts to self-quarantine and self-monitor for symptoms for 14 days from the last potential exposure date. Symptomatic travelers and contacts were offered a SARS-CoV-2 test and asked to self-isolate until either a negative test result was obtained or, following CDC recommendations at the time, until 7 days after symptom onset, including 3 days with no fever and no worsening of symptoms. Following CDC guidance at the time,* persons were considered symptomatic if they had a documented temperature of ≥100.0°F (37.8°C) or reported subjective fever, acute cough, shortness of breath, sore throat, chills, muscle aches, runny nose, headache, nausea, vomiting, diarrhea, or loss of sense of smell or taste. In addition, travelers and contacts were offered the opportunity to enroll in a home-monitoring program developed by UTHA in partnership with Sentinel Healthcare.^†^ During the contact tracing interview, data were recorded and stored in a secure, online drive.

If testing was recommended, UTHA nurses used a person-under-investigation (PUI) form to collect information on symptom status, any underlying medical conditions, and smoking status^§^ before scheduling a test. Nasopharyngeal swab specimens were collected at UTHA’s drive-through testing site. A private reference laboratory in Austin, Texas, conducted RT-PCR testing on collected samples using a cobas SARS-CoV-2 qualitative assay (Roche Molecular Systems, Inc.), which was given emergency use authorization by the Food and Drug Administration.^¶^ For those who were not residing in Austin but were recommended for testing, Austin Public Health passed on their information to the appropriate public health jurisdiction. Once a traveler or contact had a positive test result, further identification of contacts was conducted. Because of the limited number of tests available at the time, travelers and contacts were only tested once.

By March 30, nine of the first 19 travelers and contacts tested had a positive test result. Because approximately one half of persons identified and tested had a positive test result 2 days into the investigation, testing criteria were broadened to include any traveler to Cabo San Lucas, regardless of symptom status, but only symptomatic contacts continued to qualify for testing. Based on the SARS-CoV-2 incubation period of 14 days from date of exposure (*1*), the presumptive incubation period that began on March 19 when travelers returned from Cabo San Lucas ended on April 2. Therefore, after April 2, testing was only performed for exposed, symptomatic travelers and contacts. The investigation ended on April 5 when the last symptomatic contacts received negative test results.

Descriptive statistics and bivariate analyses were performed using Stata (version 16; StataCorp). Unadjusted logistic regression models were used to calculate odds ratios (ORs) and 95% confidence intervals (CIs), which were used to evaluate differences in symptoms and smoking status between persons who did and did not have positive SARS-CoV-2 test results. Because seven contacts and travelers had testing for SARS-CoV-2 performed at other sites and PUI forms were incomplete for 26, data on symptoms and underlying medical conditions are missing for 33 (14%) persons.

Among 298 persons identified during the investigation, 289 (97%) were interviewed. Contact tracing interviews revealed that Cabo San Lucas travelers used a variety of commercial, charter, and private flights to return to the United States. Although the index patient whose illness started the investigation was not symptomatic until after arriving home (March 22), other travelers experienced symptoms during March 15–19 while in Cabo San Lucas (Figure). Further, many Cabo San Lucas travelers reported prolonged exposure and reexposure to multiple other travelers because they shared hotel rooms in Mexico and apartments or other shared living spaces upon return to Austin.

**FIGURE:**
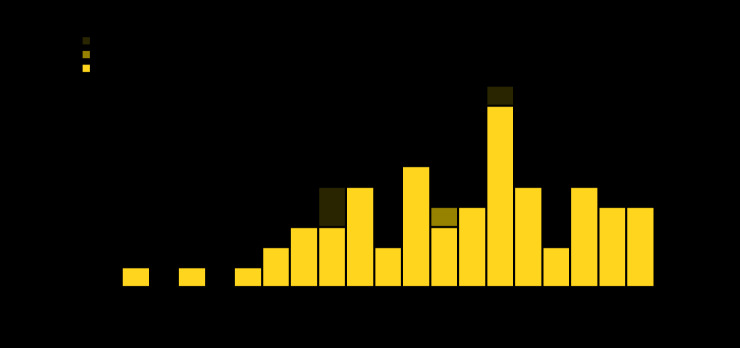
COVID-19 cases (n = 64) following a spring break trip to Cabo San Lucas, Mexico, by exposure source and date of symptom onset,* and public health investigation — Austin, Texas, March 12–April 5, 2020 **Abbreviations:** COVID-19 = coronavirus disease 2019; UT = University of Texas. * For asymptomatic cases, date of testing is used as a proxy for date of symptom onset.

Among the 231 (80%) persons tested, 183 (79%) were Cabo San Lucas travelers, and 48 (21%) were contacts of travelers with diagnosed COVID-19, including 13 (6%) household contacts and 35 (15%) community contacts (Table 1). Among all persons tested, 110 (55%) were male, and the median age was 22 years (range = 19–62 years); 179 (89%) were non-Hispanic white. The prevalence of underlying medical conditions was low (15; 8%), but nearly a quarter (45; 24%) were current smokers. Overall, 64 (28%) persons had a positive test result, including 60 (33%) of 183 Cabo San Lucas travelers, one (8%) of 13 household contacts, and three (9%) of 35 community contacts. Persons for whom testing was performed reported a median of four contacts (range = 0–15) from the 2 days preceding symptom onset (or date of testing, if asymptomatic) through their date of self-isolation. No persons were hospitalized, and none died.

**TABLE 1:** Demographic characteristics and symptoms of persons who received SARS-CoV-2 virus reverse transcription–polymerase chain reaction testing (n = 231), by contact type — Austin, Texas, March 26–April 5, 2020

Characteristic	No. (%)			
	Total n = 231 (100)	Cabo San Lucas travelers n = 183 (79)	Household contacts n = 13 (6)	Community contacts n = 35 (15)
**Age, yrs, median (range)**	22 (19–62)	21 (19–22)	22 (22–52)	22 (20–23)
Gender* (n = 202)				
Male	110 (54.5)	81 (52.3)	10 (76.9)	19 (55.9)
Female	92 (45.5)	74 (47.7)	3 (23.1)	15 (44.1)
Race/Ethnicity* (n = 202)				
White, non-Hispanic	179 (88.6)	140 (90.3)	11 (84.6)	28 (82.4)
Black, non-Hispanic	0 (0.0)	0 (0.0)	0 (0.0)	0 (0.0)
Hispanic	17 (8.4)	10 (6.5)	2 (15.4)	5 (14.7)
Other	6 (3.0)	5 (3.2)	0 (0.0)	1 (2.9)
**Positive SARS-CoV-2 test result**	64 (27.7)	60 (32.8)	1 (7.7)	3 (8.6)
**Symptomatic**	134 (58.0)	89 (48.6)	13 (100)	32 (91.4)
**Signs and Symptoms^†^ (n = 198)**				
Cough	79 (39.9)	44 (29.1)	9 (69.2)	26 (76.5)
Sore throat	64 (32.3)	44 (29.1)	5 (38.5)	15 (44.1)
Headache	43 (21.7)	25 (16.6)	5 (38.5)	13 (38.2)
Loss of smell or taste (n = 215)	37 (17.2)	26 (14.8)	3 (27.3)	8 (28.6)
Shortness of breath	28 (14.1)	13 (8.6)	4 (30.8)	11 (32.4)
Muscle aches	27 (13.6)	15 (9.9)	3 (23.1)	9 (26.5)
Diarrhea	25 (12.6)	20 (13.3)	1 (7.7)	4 (11.8)
Chills	18 (9.1)	12 (8.0)	0 (0.0)	6 (17.7)
Fever	19 (9.6)	10 (6.6)	1 (7.7)	8 (23.5)
Abdominal pain	9 (4.6)	5 (3.3)	1 (7.7)	3 (8.8)
Vomiting	4 (2.0)	3 (2.0)	0 (0.0)	1 (2.9)
Other	38 (19.2)	21 (13.9)	5 (38.5)	12 (35.3)
**Underlying medical conditions^§^ (n = 192)**				
Chronic lung disease	9 (4.7)	6 (4.0)	1 (8.3)	2 (6.5)
Immunocompromised	4 (2.1)	2 (1.3)	1 (8.3)	1 (3.2)
Hypertension	2 (1.0)	1 (0.7)	1 (8.3)	0 (0.0)
Cardiovascular disease	2 (1.0)	1 (0.7)	0 (0.0)	1 (3.2)
Diabetes	0 (0.0)	0 (0.0)	0 (0.0)	0 (0.0)
Chronic kidney disease	0 (0.0)	0 (0.0)	0 (0.0)	0 (0.0)
Chronic liver disease	0 (0.0)	0 (0.0)	0 (0.0)	0 (0.0)
Pregnancy	0 (0.0)	0 (0.0)	0 (0.0)	0 (0.0)
**Smoking status^¶^ (n = 191)**				
Current smoker	45 (23.6)	31 (20.9)	6 (50.0)	8 (25.8)
Former smoker	20 (10.5)	13 (8.8)	1 (8.3)	6 (19.4)
Never smoked	126 (66.0)	104 (70.3)	5 (41.7)	17 (54.8)

* The number of available responses for gender and race/ethnicity is 202 (12.6% missing), with 155 (15.3% missing) for Cabo San Lucas travelers, 13 (0.0% missing) for Household contacts, and 34 (2.9% missing) for Community contacts.

^†^ The number of available responses for signs and symptoms, with the exception of loss of sense of taste and smell, is 198 (14.3% missing), with 151 (17.5% missing) for Cabo San Lucas travelers, 13 (0.0% missing) for Household contacts, and 34 (2.9% missing) for Community contacts. Loss of sense of taste or smell was evaluated by both contact tracers and triage nurses, resulting in 215 available evaluations (6.9% missing), with 176 (3.8% missing) for Cabo San Lucas travelers, 28 (20% missing) for Community contacts, and 11 (6.9% missing) for Household contacts.

^§^ The number of missing responses for underlying medical conditions is 39 (16.9% missing), with 149 (18.6% missing) for Cabo San Lucas travelers, 12 (7.7% missing) for Household contacts (7.7%), and 31 (11.4% missing) for Community contacts.

^¶^ The number of available responses for smoking status is 191 (17.3% missing), with 148 (19.1% missing) for Cabo San Lucas travelers, 12 (7.7% missing) for Household contacts, and 31 (11.4% missing) for Community contacts.

Among the 64 persons with positive SARS-CoV-2 RT-PCR test results, 14 (22%) were asymptomatic and 50 (78%) were symptomatic at the time of testing (Table 2). Among those who had a positive test result, the most commonly reported symptoms were cough (21; 38%), sore throat (18; 32%), headache (14; 25%), and loss of sense of smell or taste (15; 25%); only six (11%) reported fever. Among persons with negative test results, 84 (50.3%) reported symptoms; the most commonly reported symptoms were cough (58; 41%), sore throat (46; 32%), headache (29; 20%), and loss of sense of smell or taste (22; 14%); 13 (9%) reported fever. The odds of having a positive test result were significantly higher among those who were symptomatic than among those who were asymptomatic (OR = 3.5; 95% CI = 1.8–7.4). There were no significant differences in the types of symptoms reported among persons with positive and negative test results, nor were there any significant differences in smoking status among persons with positive and negative test results.

**TABLE 2:** Association of symptom status and symptoms reported among persons who received SARS-CoV-2 virus reverse transcription–polymerase chain reaction testing (n = 231) — Austin, Texas, March 26–April 5, 2020

Characteristic	No. (%)		
	Positive test (n = 64)	Negative test (n = 167)	Unadjusted odds ratio (95% CI)
**Symptom status**			
Asymptomatic	14 (21.9)	83 (49.7)	Ref
Symptomatic	50 (78.1)	84 (50.3)	3.53 (1.75–7.42)
Symptoms (n = 198)*			
Cough	21 (37.5)	58 (40.9)	0.87 (0.46–1.64)
Sore Throat	18 (32.1)	46 (32.4)	0.99 (0.51–1.92)
Headache	14 (25.0)	29 (20.4)	1.30 (0.63–2.70)
Loss of smell or taste (n = 215)	15 (24.6)	22 (14.3)	1.96 (0.94–4.09)
Chills	8 (14.3)	10 (7.0)	2.20 (0.82–5.90)
Diarrhea	8 (14.3)	17 (12.0)	1.23 (0.50–3.03)
Fever	6 (10.7)	13 (9.2)	1.19 (0.43–3.31)
Shortness of breath	4 (7.1)	24 (16.9)	0.38 (0.12–1.14)

* The number of available responses for symptoms, except for loss of smell or taste, is 198 (14.3% missing), with 56 (12.5% missing) for positive test results and 142 (15.0% missing) for negative test results. Loss of sense of taste or smell was evaluated by both contact tracers and triage nurses, resulting in 215 available evaluations (6.9% missing), with 61 (4.7% missing) for positive test results and 154 (7.8%) for negative test results. The reference group for the logistic regressions that examined the association of specific symptoms with test results is those persons who tested negative.

## Public Health Response

The UTHA COVID-19 Center, a novel university–public health partnership established with the local public health entity, Austin Public Health, led the outbreak response. During the early stage of the pandemic in March, resources among institutions were pooled to improve the capacity to identify and interview a large number of travelers and contacts, to facilitate testing, and to follow travelers and contacts. University Health Services coordinated additional support for students’ housing, food, and other needs during isolation and quarantine.

In addition, concurrent actions at the university level and across Austin aimed at limiting COVID-19 spread in the community were undertaken, including rapid contact tracing, a municipal shelter-in-place order on March 25 (Figure), the university’s extension of spring break by a week, and a transition to remote learning when operations resumed on March 30. Austin Public Health and University of Texas Austin publicized the ongoing investigation on March 31 and April 3, respectively, and encouraged community members to avoid nonessential travel and seek testing if they had symptoms. UTHA also provided updates about the ongoing investigation to the UTHA community through email.

## Discussion

Investigation of an outbreak of COVID-19 among a group of college-aged travelers and their contacts demonstrated that 28% had positive SARS-CoV-2 RT-PCR test results, approximately one fifth of whom were asymptomatic when tested. Asymptomatic transmission has been documented in multiple settings and has led to large outbreaks (*2*–*6*). Asymptomatic persons or those with mild symptoms likely play an important role in sustaining SARS-CoV-2 transmission during outbreaks, especially in younger populations, such as the one described here. The high prevalence of asymptomatic persons underscores the importance of testing both symptomatic and asymptomatic persons after a known COVID-19 exposure.

No constellation of symptoms was diagnostic of COVID-19 in this population. Similar proportions of fever, cough, sore throat, and headache occurred among persons with positive test results and those with negative results. Because testing supplies were limited, only symptomatic persons were tested during March 28–30. Some persons might have reported symptoms as a means to get tested during that time. A possibility also exists that a separate, concomitant respiratory illness occurred among travelers and contacts in March that might explain the similarities in symptoms between those who had positive test results and those who had negative results. Although persons with negative SARS-CoV-2 test results in this analysis were not tested for influenza or other respiratory illnesses, widespread transmission of influenza was reported by the U.S. Department of Health and Human Services during March 8–March 21.** Recent studies have demonstrated variability in symptoms such that strict implementation of guidance that emphasizes a symptom-based approach to COVID-19 testing could result in missing a diagnosis of COVID-19 in a sizeable proportion of cases (*7*,*8*).

During contact tracing interviews, Cabo San Lucas travelers reported sharing housing in both Mexico and upon return to Austin. The proximity created by this shared housing likely contributed to transmission through ongoing exposure and reexposure to SARS-CoV-2. This pattern of social interaction, in which residents gather frequently to socialize and share facilities, is common among many college-aged persons and might lead to propagated spread, similar to the continued person-to-person transmission observed in long-term care facilities (*5*). The prevalence of shared housing and prolonged exposure experienced by the college-aged Cabo San Lucas travelers highlights the importance of universities and schools considering how to align students’ living arrangements with CDC recommendations for living in shared housing^††^ as they plan to reopen.

The findings in this report are subject to at least five limitations. First, the majority of students were only tested for SARS-CoV-2 once because of limited test availability at the time; therefore, some asymptomatic or presymptomatic cases might have been missed. Second, seven travelers and contacts did not reside in Austin and were tested elsewhere. For these seven, investigators relied upon self-reported test results, and information on demographic characteristics and symptoms was not available. Third, a number of PUI forms had missing information regarding demographic characteristics, symptoms, or underlying health conditions. Although it is possible that the missing information regarding symptoms and underlying health conditions could influence the prevalence of symptoms seen in this investigation, the variability of reported signs and symptoms is consistent with what has been published in recent literature (*7*,*8*). Fourth, the diagnostic sensitivity of the RT-PCR test used is not yet known. Although this particular RT-PCR test demonstrates an analytic sensitivity of 95% at concentrations of 46 copies of virus/mL, the first systematic reviews suggest that similar RT-PCR tests are demonstrating a false-negative rate of 2%–29%^§§^ (*9*). Finally, the significant overlap between students who went on the trip together and those who shared living quarters after returning to Austin made it difficult to estimate accurate primary and secondary infection rates.

As schools and universities make decisions about reopening, it is important that they plan for isolating and testing persons with suspected COVID-19, quarantining their contacts, and implementing suggestions described in CDC’s Considerations for Institutes of Higher Education.^¶¶^ Coordination between educational institutions and health authorities can facilitate rapid identification of cases, contact tracing, active surveillance, and identification of clusters. Contact tracing and testing of close contacts, regardless of symptoms, is important in limiting spread, especially in young and healthy populations living in shared housing and in controlling future COVID-19 outbreaks that might occur as schools and universities consider reopening.

**Summary**

**What is already known about this topic?**

COVID-19 can cause asymptomatic and mild illness, particularly among young, healthy populations.

**What is added by this report?**

Transmission of SARS-CoV-2 during and after a college spring break trip (March 14–19) led to 64 cases, including 60 among 183 vacation travelers, one among 13 household contacts, and three among 35 community contacts. Prompt epidemiologic investigation, with effective contact tracing and cooperation between a university and a public health department, contributed to outbreak control.

**What are the implications for public health practice?**

A coordinated response with contact tracing and testing of all contacts, including those who are asymptomatic, is important in controlling future COVID-19 outbreaks that might occur as schools and universities consider reopening.

## Footnotes

* https://emergency.cdc.gov/han/2020/HAN00429.asp.

† https://sentinel.healthcare/2020/04/07/sentinel-healthcare-announces-partnership-with-ut-health-austin-to-launch-quarantine-management-platform-for-novel-coronavirus/.

§ Smoking status includes reported use of either combustible cigarettes, e-cigarettes, or both.

¶ The clinical diagnostic sensitivity investigations are ongoing, but analytical sensitivity studies that compare the cobas SARS-CoV-2 qualitative assay against an authorized RT-PCR test using a symptomatic patient’s SARS-CoV-2 virus specimen demonstrate a sensitivity of 95% at concentrations as low as 46 virus copies/mL. https://www.fda.gov/media/136049/download.

** https://www.dshs.state.tx.us/idcu/disease/influenza/surveillance/2019-2020.aspx.

†† https://www.cdc.gov/coronavirus/2019-ncov/daily-life-coping/shared-housing/index.html.

§§ https://www.medrxiv.org/content/10.1101/2020.04.16.20066787v1.

¶¶ https://www.cdc.gov/coronavirus/2019-ncov/community/colleges-universities/considerations.html.
